# Comprehensive Proteomics Analysis of In Vitro Canine Oviductal Cell-Derived Extracellular Vesicles

**DOI:** 10.3390/ani11020573

**Published:** 2021-02-23

**Authors:** Seok Hee Lee, Saúl Lira-Albarrán, Islam M Saadeldin

**Affiliations:** 1 Center for Reproductive Sciences, Department of Obstetrics, Gynecology and Reproductive Sciences, University of California, San Francisco, San Francisco, CA 94143, USA; saul.liraalbarran@ucsf.edu; 2 Department of Physiology, Faculty of Veterinary Medicine, Zagazig University, Zagazig 44519, Egypt; 3 Department of Animal Production, College of Food and Agriculture Sciences, King Saud University, Riyadh 11451, Saudi Arabia; 4 King Faisal Specialist Hospital & Research Centre, Department of Comparative Medicine, Riyadh 11211, Saudi Arabia

**Keywords:** canine oviduct, exosomes, extracellular vesicles, proteomics

## Abstract

**Simple Summary:**

As the dog shows unique and peculiar reproductive characteristics, assisted reproductive techniques such as in vitro maturation and in vitro fertilization have not been well-established compared with those of other mammals. Our recent work demonstrated the interplay between in vitro oviductal cell-derived extracellular vesicles (OC-EVs) and cumulus-oocyte complexes in dogs. Here, we provided for the first time a comprehensive proteomic analysis of OC-EVs. A total of 398 proteins were identified in all OC-EVs samples. A functional enrichment analysis indicated that these core proteins were involved in the key cellular metabolic process related to oocyte maturation and embryonic development. The current comprehensive description of the canine OC-EVs proteome would provide a fundamental resource for further understanding canine reproductive physiology, the interaction of sperms with female counterparts during fertilization, early pregnancy, and establishing an efficient system of in vitro embryo production.

**Abstract:**

Dogs (Canis lupus familiaris) have unique and peculiar reproductive characteristics. While the interplay between in vitro oviductal cell-derived extracellular vesicles (OC-EVs) and cumulus-oocyte complexes in dogs has begun to be elucidated, no study has yet provided extensive information on the biological content and physiological function of OC-EVs and their role in canine oocyte development. Here, we aimed to provide the first comprehensive proteomic analysis of OC-EVs. We identified 398 proteins as present in all OC-EVs samples. The functional enrichment analysis using Gene Ontology terms and an Ingenuity Pathway Analysis revealed that the identified proteins were involved in several cellular metabolic processes, including translation, synthesis, expression, and protein metabolism. Notably, the proteins were also involved in critical canonical pathways with essential functions in oocyte and embryo development, such as ERK/MAPK, EIF2, PI3K/AKT, and mTOR signaling. These data would be an important resource for studying canine reproductive physiology and establishing a successful in vitro embryo production system in dogs.

## 1. Introduction

Cells release different types of extracellular vesicles (EVs) in the extracellular microenvironment [1]. They affect recipient cells directly through the transfer of bioactive cargo (mRNA, proteins, and lipids) or indirectly through affecting the cellular epigenome [2,3]. EVs and exosomes have been isolated from various types of cells and biological fluids such as saliva [4], blood plasma [5], and urine [6]. Concerning reproductive fluids, they can be obtained from the uterine [7], seminal [8], follicular [9], and oviductal fluids [10]. Given their ubiquitous role, it has been proposed that EVs and exosomes isolated from reproductive samples are closely related to gamete and embryo development [11,12]. In 2013, Al-Dossary et al. dubbed the exosomes derived from the oviductal fluid as “oviductosomes” [13] based on their site of origin. There is markedly increasing interest in understanding oviduct-derived EVs for their potential physiological roles in the reproductive process, such as sperm capacitation, oocyte maturation, and embryo development [10,14,15].

Our recent studies have demonstrated that canine in vitro oviductal cell-derived extracellular vesicles (OC-EVs) affect not only the viability, proliferation rate, and gene/protein expression of cumulus cells [16] but, also, exert regulatory functions on cumulus–oocyte complexes (COCs) by enhancing oocyte development via the EGFR/MAPK signaling pathway [17]. Moreover, the proteins derived from oviductal EVs could regulate the physiological functions of gamete and embryos [18,19].

Previous proteomic research demonstrated the protein composition of the oviductal fluid. Those proteomes have been suggested as a potential tool for understanding reproductive physiology [20,21,22,23,24]. However, few systematic studies are unraveling the molecular content of the OC-EVs to understand their possible roles in gamete/oocyte/embryo development in the canine reproductive system. Therefore, this study would provide valuable information regarding the protein content and its molecular function with the signaling pathway in OC-EVs.

The proteomic content of oviductal EVs has been studied in different species. For example, mouse oviductal fluid contains plasma membrane Ca^2+^-ATPase 4a and epididymal sperm adhesion molecule 1, molecules that play an essential role in sperm capacitation and fertility [15,25]. Similarly, in bovines, 319 proteins were identified in EVs from the oviduct; several of these proteins were involved in fertilization and embryo development [10].

In this study, we aimed to describe the proteome of canine OC-EVs. This endeavor is essential, given the unique reproductive characteristics of bitches compared with other mammals: at ovulation, the oocyte is in prophase I and will undergo maturation into a metaphase II in the oviductal canal after a period of 48–72 h [26,27]. Therefore, understanding the protein composition of canine OC-EVs can provide valuable information for the establishment of a successful in vitro maturation system.

Therefore, our efforts in this research were directed towards characterizing canine OC-EV protein compositions by employing liquid chromatography-tandem mass spectrometry (LC-MS/MS) and its potential physiological relevance following a functional analysis of the resultant set of proteins. This comprehensive study in canine species will form a platform to suggest the potential role of EVs in canine oocyte development and bring new insight into the EV contributions to establishing stable assisted reproductive techniques in canine reproduction.

## 2. Materials and Methods

### 2.1. Chemical

The chemicals used in this study were obtained from Sigma-Aldrich Co., LLC. (St. Louis, MO, USA) unless otherwise stated.

### 2.2. Collection of Conditioned Medium and Isolation of Canine In Vitro Oviductal Cell-Derived Extracellular Vesicles

In the present study, we collected the OC-EVs from oviduct cells that were obtained from our previous research [16,17,28,29,30]. Each sample was isolated from different individuals, and the cryopreserved in vitro oviduct cells maintained their epithelial characteristics (positive for cytokeratin) after thawing, as described in our previous study [17]. In this study, oviduct cells from three different individuals were used for collecting OC-EVs. Briefly, the cryopreserved canine oviduct cells were thawed and cultured at the same volumes of medium containing 10% fetal bovine serum (FBS) with 1 μg/mL of progesterone (P4) for 24 h. The medium was then exchanged with the same volumes of medium containing exosome-depleted FBS (System Biosciences, San Francisco, CA, USA) with 1 μg/mL of P4 and cultured for an additional 24 h. Then, the supernatant was retrieved and centrifuged for 30 min at 2000× *g* at 4 °C. The Total Exosome Isolation Reagent (Life Technologies, Carlsbad, CA, USA) was used for collecting OC-EVs using the manufacturer’s instructions. First, the cell supernatant was centrifuged at 2000× *g* for 30 min at room temperature to remove any cells and debris. After that, the supernatant was transferred into a new sterilized tube without disturbing the pellets, and the Total Exosome Isolation Reagent was added proportionally (1:1) to the volume of supernatant using the manufacturer’s instructions. The mixtures were vortexed and incubated at 4°C overnight. The sample was centrifuged at 10,000× *g* for 1 h, and the supernatant was discarded without disturbing the exosomal pellets. The pellets were stored at 4 °C until further proteomic experiments.

### 2.3. Characterization of Canine In Vitro Oviductal Cell-Derived Extracellular Vesicles

The morphology and size of the OC-EVs was evaluated by transmission electron microscopy, as previously described [16,17]. Briefly, the OC-EVs were obtained from 10 mL of the culture medium, and the samples were diluted in 200 μL of nuclease-free water. The suspensions were then transferred to copper 200-mech Formvar-coated carbon stabilized grids and allowed to adsorb to the grid for 4 to 5 min. After wiping out the suspensions with filter paper, 5 μL of 1% aqueous uranyl acetate was applied to the grid to stain the EVs for 30 sec, and then, the staining reagent was wiped out with filter paper. After rinsing out the grids with drops of deionized water (3 times for 10 sec each), the samples were allowed to air dry for 5 min. Finally, the analysis was performed using a LIBRA 120 transmission electron microscope (Carl Zeiss, Oberkochen, Germany) at 110kV. The concentration, size, and intensity of the oviduct-derived EVs was evaluated by NTA (Nanosight LM10, Malvern, UK). In brief, the purified EVs were diluted in ~1-mL phosphate-buffered saline, and then, the mean, mode, standard error of the mean, and concentration of particles were recorded by NTA 2.3 software (Nanosight LM10, Malvern, UK). The concentration of particles was adjusted to achieve ~50 vesicles in one screen to obtain appropriate counting for quantification. With the identical system setting values, the measurements of the EVs were performed.

### 2.4. Preparation of Canine In Vitro Oviductal Cell-Derived Extracellular Vesicles Protein Fraction by Sodium Dodecyl Sulfate-Polyacrylamide Gel Electrophoresis and in-Gel Tryptic Digestion

For the preparation of the extracellular protein fraction, the pellets containing OC-EVs were suspended in 20-mM Tris-HCl (pH 8.0, 100 μL). The OC-EVs were incubated in a denaturation buffer containing 2% sodium dodecyl sulfate (SDS) and 25-mM ammonium bicarbonate for 1 h at room temperature. Then, the mixture was centrifuged for 10 min at 18,000 rpm to remove cell debris. The protein concentration was determined by using the bicinchoninic acid method. The 20 μg of crude protein mixtures of the OC-EVs were fractionated by 12% sodium dodecyl sulfate-polyacrylamide gel electrophoresis (SDS-PAGE) before protein identification (Figure S1). Tryptic digestion for the MS/MS analysis was performed as previously described [31]. According to the molecular weight, SDS-polyacrylamide gels were divided into a total of ten fragments. Sliced gels were destained with a solution containing 50% acetonitrile and 10-mM ammonium bicarbonate. Then, the gels were washed with distilled water, followed by 100% acetonitrile. A reducing solution composed of 10-mM dithiothreitol was treated to the remaining proteins in each gel, and an alkylation solution containing 55-mM iodoacetamide was added to break the disulfide bonds in the proteins. After washing the gels with distilled water, the fragments of the gels were digested with trypsin (Promega, Madison, WI, USA) at 37 to 38 °C for 16 h. Then, extraction of the digested peptides was performed with an extraction solution (50-mM ammonium bicarbonate and 50% acetonitrile containing 5% trifluoroacetic acid (TFA)). The final extracts were lyophilized, and the samples were dissolved in 0.5% TFA for LC-MS/MS.

### 2.5. Protein Identification with Liquid Chromatography with Tandem Mass Spectrometry Analysis

Digested peptide samples (10 μL) were concentrated using an MGU-30 C18 trapping column (LC Packings, Amsterdam, The Netherlands) and eluted from the column. Then, the concentrated tryptic peptides were directed onto a C18 reverse-phase column (10 cm × 5 mm I.D.; Proxeon Biosystems, Odense, Denmark) at a flow rate of 120 nl/min. Peptides were eluted by a gradient of 0–65% acetonitrile for 100 min. All MS and MS/MS spectra were acquired in a data-dependent mode with an LTQ-Velos electrospray ionization Ion Trap mass spectrometer (Thermo Scientific, Dreieich, Germany). Three MS/MS scans of the most abundant precursor ions with the dynamic exclusion feature enabled were selected from each full MS (*m/z* range 400–2000) scan. For protein identification, MS/MS spectra of at least one peptide were analyzed by MASCOT v2.4 (Matrix Science, Inc., Boston, MA, USA). Tolerance of the oxidation of methionine, carbamidomethylation of cysteines, two missed trypsin cleavages, and the peptide was 0.8 Da, and mass tolerance of the fragment was 0.8 Da for searching parameters. The genome sequence database was downloaded from the National Center for Biotechnology Information and used for protein identification. The mol% was calculated by using an exponentially modified protein abundance index (emPAI) generated by MASCOT. The MS/MS analysis was performed at least three times for each sample, and the MS/MS data were filtered according to a false discovery rate (FDR) criterion of 1%.

### 2.6. Bioinformatic Analysis for the Characterization of Identified Canine In Vitro Oviductal Cell-Derived Extracellular Vesicles Proteins by Proteomic Methods

A Venn diagram (https://bioinformatics.psb.ugent.be/webtools/Venn (accessed on 14 February 2020)) was created by combining the gene proteins of each of the three canine biological samples to identify the common proteins of the OC-EVs within these samples. The overrepresentation analysis (Fisher’s exact test) of the common proteins identified was performed using PANTHER (version 15.0 released 14 February 2020) with the FDR correction method by applying a significance threshold of FDR < 0.05 [32]. The overrepresented GO terms, biological processes (BPs), molecular functions (MFs), and cellular components (CCs) were summarized with REVIGO (reduce + visualize Gene Ontology, http://revigo.irb.hr (accessed on 14 February 2020)) to avoid redundant GO terms [33]. The nomenclature of the BPs, MFs, and CCs used the terms of the Gene Ontology Consortium [34]. The core analysis generated with the Ingenuity Pathway Analysis software (IPA; http://ingenuity.com (accessed on 14 February 2020)) identified the biofunctions and canonical pathways (*p*-value < 0.01, using the right-tailed Fisher’s exact test), as well as the networks using the list of common proteins from three biological samples of canine OC-EVs. Additionally, the molecular activity predictor analysis was used to identify the relevant molecules associated with particular biofunctions based on a hypothesis-driven approach.

## 3. Results

### 3.1. Characterization of In Vitro Oviductal Cell-Derived Extracellular Vesicles

**Figure 1:**
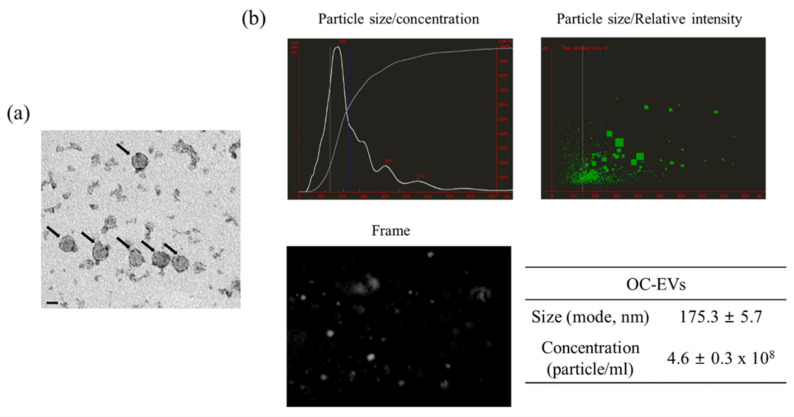
Characterization of canine OC-EVs. (**a**) Morphological characterization of EVs isolated from in vitro cultured oviduct cells using transmission electron microscopy (Scale bar = 100 nm). Black arrows indicate EVs. (**b**) Characterization of EVs regarding particle size, concentration, and relative intensity using a nanoparticle tracking analysis (NTA). Data are shown as means ± standard error of the mean. OC-EVs: canine in vitro oviductal cell-derived extracellular vesicles.

OC-EVs were obtained using the well-established methodology described in our previous research [16,17]. We confirmed that OC-EVs have a spherical shape of 150–180 nm in diameter (Figure 1a). A nanoparticle tracking analysis (NTA) identified particles 175.3 ± 5.7 nm in size with concentrations of 4.6 ± 0.3 × 10^8^ particles/mL (Figure 1b). The previously published work from our group showed that exosomal-specific markers (CD9, CD81, and CD63) were expressed in OC-EVs [16,17]. Besides, the absence of the non-exosomal-specific protein (calnexin) was confirmed in the samples in the previous study. Therefore, these data indicated that the successful isolation and purification of canine OC-EVs could be further applied to a proteomic analysis.

### 3.2. Functional Enrichment Analysis of Common Proteins Identified in Canine In Vitro Oviductal Cell-Derived Extracellular Vesicles

#### 3.2.1. Gene Ontology Analysis for Canine In Vitro Oviductal Cell-Derived Extracellular Vesicle Proteomes

**Figure 2:**
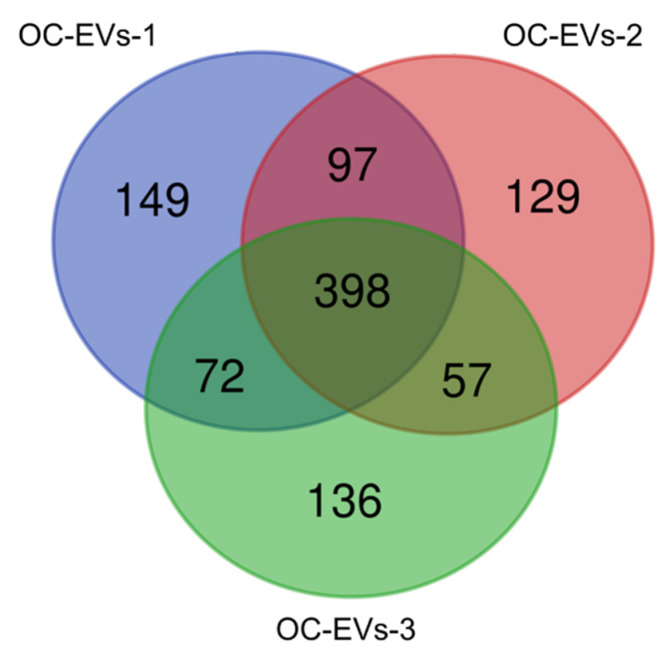
Numbers of proteins identified in the samples derived from the canine in vitro oviductal cell-derived extracellular vesicles. Venn diagram showing 398 shared proteins between three biological samples of canine in vitro oviductal cell-derived extracellular vesicles.

A comprehensive LC-MS/MS proteomic analysis was performed to evaluate the OC-EV protein compositions. A total of 1038 proteins among the three groups were identified (Table S1). The number of shared proteins identified in the OC-EVs of the three biological samples evaluated was 398 (38.3% of the total) (Figure 2 and Table S2).

**Figure 3:**
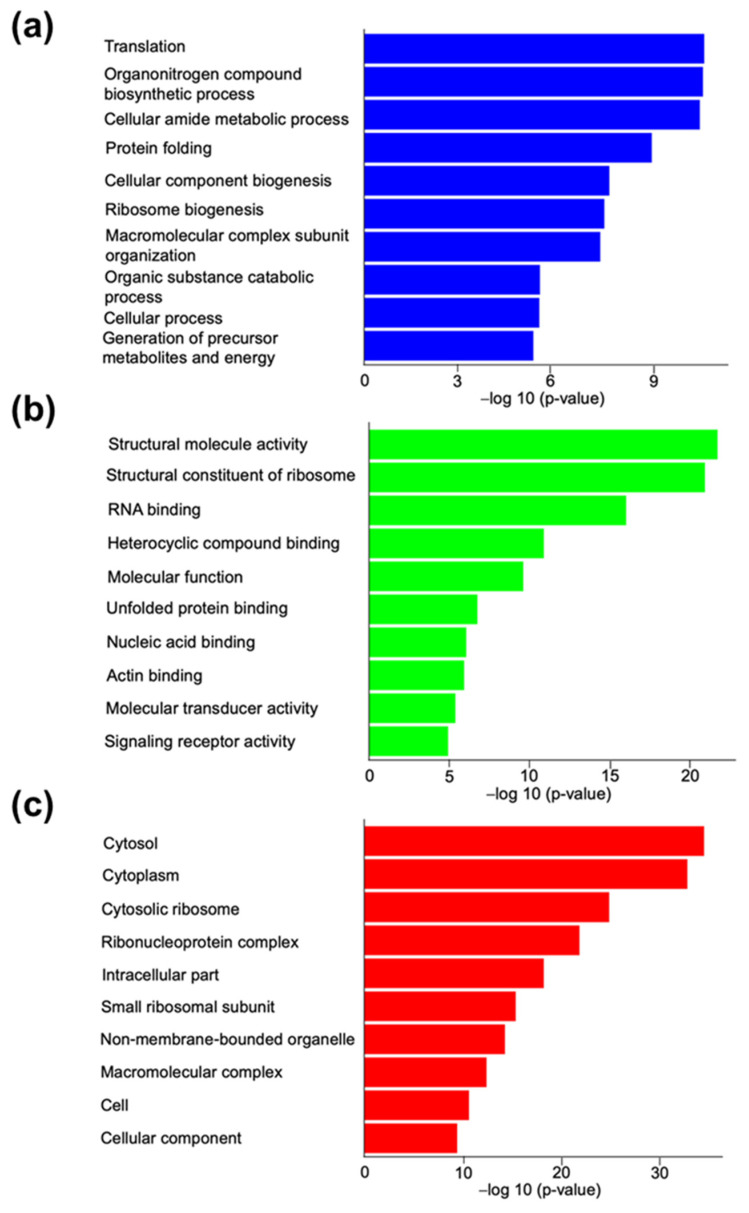
Parental Gene Ontology terms from a proteomic analysis for canine in vitro oviductal cell-derived extracellular vesicles. The top ten (**a**) biological processes, (**b**) molecular functions, and (**c**) cellular components are overrepresented using the list of 398 common proteins identified in three biological samples of canine in vitro oviductal cell-derived extracellular vesicles.

Importantly, from those samples, several EV marker proteins, including heat shock protein (HSP) 70, HSP90, and cytosolic proteins (annexins and Ras-related proteins), were identified. In contrast, calnexin (an intracellular protein used as a negative marker of EVs) was not identified in the analysis, consistent with our previous Western blot results [17]. We describe the top 20 of the identified 398 shared proteins from the OC-EVs in Table S3, and the protein with the highest percentage of relative abundance in these samples was vimentin. A Gene Ontology (GO) analysis was conducted to gain insight into the potential physiological relevance of EV proteins. This analysis identified 169 biological processes (BPs; Table S4A) highlighting 84 parental BPs (Table S4B), 54 molecular functions (MFs; Table S4C), including 42 parental MFs (Table S4D), and 65 cellular components (CCs; Table S4E) also highlighting 42 parental CCs (Table S3F) using the list of all 398 shared proteins from the OC-EVs. The top 10 nonredundant GO terms from these three categories are shown in Figure 3.

The GO analysis highlighted the physiological relevance of the OC-EVs proteins in the metabolism. In addition, the overrepresentation analysis by PANTHER identified several metabolic pathways, such as the tricarboxylic acid cycle, pentose phosphate pathway, and ATP synthesis (Table S4G). The classes of proteins defined by PANTHER with the highest fold enrichment were hydratase, Hsp90 family chaperone, and chaperonin (Table S4H).

#### 3.2.2. Ingenuity Pathway Analysis for Canine In Vitro Oviductal Cell-Derived Extracellular Vesicle Proteomes

To highlight the specific biological processes underpinning the function of the OC-EVs, the Ingenuity Pathway Analysis (IPA) was used to identify the novel biofunctions, canonical pathways, and network of these enriched OC-EV core proteins. We performed a core analysis using the dataset of 398 shared proteins against all identified 1038 proteins (Table S2). The core analysis using the list of 398 shared proteins in the OC-EV proteomes identified 500 biofunctions, 120 canonical pathways, and 18 statistically significant networks enriched with OC-EV core proteins (Table S5).

The main biofunctions were the initiation of translation of the protein, the decay of mRNA, and the metabolism of the protein (Table 1 and Table S5A), giving support to our previous GO analysis. The most statistically significant canonical pathways included EIF2 and the regulation of elF4 and p70S6K signaling (Table 2 and Table S5B), both involved in protein synthesis. In this sense, the network with the highest number of molecules identified (31/398) was associated with protein synthesis and RNA damage and repair, as well as RNA post-transcriptional modification (Table S5).

Next, the representative 120 canonical pathways (*p*-value < 0.01) and the top ten of canonical pathways were provided in Table S5B and Table 2, respectively.

**Figure 4:**
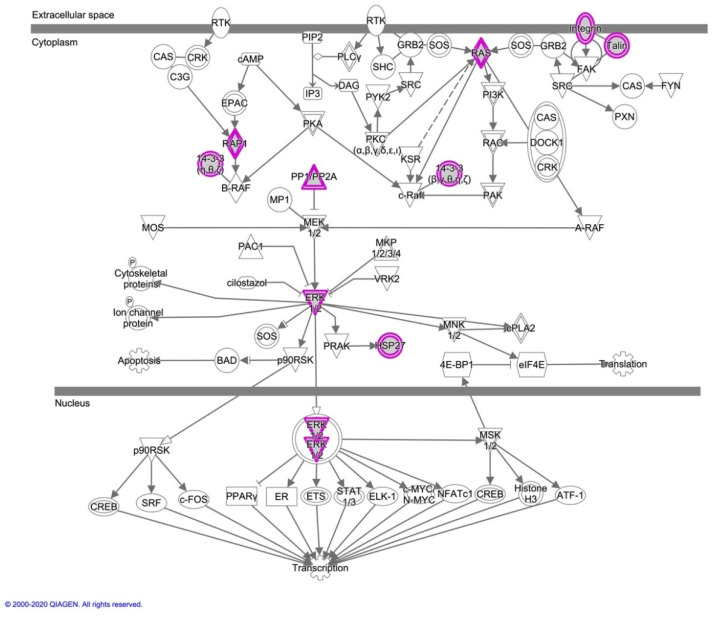
Common proteins identified in the three replicates of canine in vitro oviductal cell-derived extracellular vesicles that participate in the canonical pathway (CP) ERK/MAPK signaling. The Ingenuity Knowledge Base defines this CP. The grey molecules highlighted with pink were identified in three biological samples of canine in vitro oviductal cell-derived extracellular vesicles. The arrows represent the directionality of the interaction between molecules or a molecule and a bio-function. The vertical tip represents inhibition. The solid line is for direct interaction, and the dashed line is for indirect interaction.

The key canonical pathways essential to oocyte maturation, folliculogenesis, and embryo development were identified, including ERK/MAPK, EIF2, PI3K/AKT, and mTOR signaling (Table 2 and Table S5B). In this regard, Figure 4 and Figure S2 show the canonical pathways ERK/MAPK signaling and EIF2 signaling, respectively, highlighting in grey color the common proteins identified in OC-EVs.

**Figure 5:**
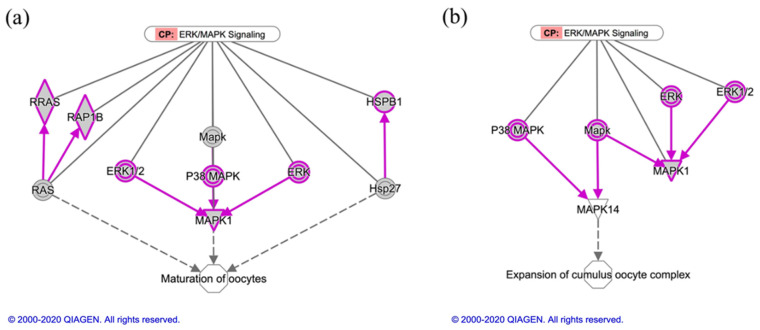
Molecules associated with the canonical pathway (CP) ERK/MAPK signaling in the function of two stages of oocyte development. The Ingenuity Knowledge Base defines both CPs. (**a**) The relationship between MAPKs, RAS, and Hsp27 with oocyte maturation in ERK/MAPK signaling. (**b**) The relationship between MAPKs with the expansion of the cumulus–oocyte complex in ERK/MAPK signaling. The proteins highlighted with shadowed blocks are identified in three biological samples of canine in vitro oviductal cell-derived extracellular vesicles. The solid gray lines represent the genes associated with a CP. The arrows represent the directionality of the interaction between molecules or a molecule and a bio-function. The solid line is for direct interaction, and the dashed line is for indirect interaction.

The OC-EVs are involved in distinguishable functions of core proteins. For example, the molecule activity predictor analysis identified several proteins that belong to the canonical pathway (ERK/MAPK signaling) and participate in oocyte maturation and cumulus cell expansions (Figure 5).

As described in Figure 5a, multiple factors, including MAPK1, ERK1/2, P38 MAPK, RAS, and HSP27, are involved in oocyte maturation [35,36,37,38]. Concerning the expansion of the cumulus–oocyte complex, P38 MAPK, MAPK1, and ERK1/2 derived from OC-EVs are involved in this process (Figure 5b), which is consistent with our recent findings [16,17].

## 4. Discussion

Canine oocyte maturation possesses a unique event in which ovarian follicles release immature prophase I oocytes, requiring an additional 48–72 h to undergo maturation in the oviductal canal [26,27,32]. The interaction between the oviduct secretome and oocytes is pivotal to the meiotic and cytoplasmic maturation of the oocytes. Hence, as a continuation of our previous studies, we characterized the OC-EVs and analyzed their protein contents to better understand their involvement in the oocyte maturation process in this unique species. The current results dig into the pathways controlled through the OC-EVs to regulate the canine oocyte maturation and early embryo development. Several studies have been reported on the molecular cargo of the oviductal EVs in different species; however, there is a lack of information in canine species.

In bovines, a mass spectrometry analysis identified 319 proteins in the oviductal EVs, where 97 proteins were exclusively expressed in in vivo EVs, 47 proteins were expressed only in vitro, and 175 proteins were common [10]. A functional analysis of the resultant proteins revealed essential pathways involved in sperm–oocyte binding and fertilization [10]. Additionally, a mass spectrometry and DAVID functional annotation clusters analysis identified 336 clusters of proteins in bovine oviductal EVs (170 were differentially abundant across the estrous cycle) that suggested the involvement of the proteins in metabolism and gamete–oviduct interactions [39]. Furthermore, a shotgun proteomics and bioinformatics analysis identified the proteome of bovine oviductal fluid and revealed 266 secreted proteins (109 (41%) of them were shared for both in vivo and in vitro conditions). Our LC-MS/MS results showed a total of 1038 proteins in the three biological samples, sharing 398 common proteins. In fact, the qualitative proteomics of EV cargoes are highly variable in both biological and technical replicates, with a higher incidence among the former. We observed 40% identical proteins in our three samples, a number within the range (35–60%) of overlapped peptide lists from pairs of technical replicates [40]. Tiruvayipati et al. [41] recently reported that only 17% could be detected as common proteins within the same cell line, which is very smaller than what we detected (~40%). Moreover, they found that there is an average variance (i.e., relative standard deviation) between the quantitative protein analysis within the same line up to 47%. Furthermore, the issue of interbiological (47%) and intrabiological variations (45%) was recently highlighted within the urinary-derived EVs [42]. Indeed, LC-MS is regarded as a highly complex analytical technique, and the proteomics experiments based on this technique can be subject to a large variability despite recent advances in technological and computational tools [43]. Therefore, future studies with larger sample sizes are required to facilitate more accurate estimations among biological variations and to reduce the biological variability among the samples.

The current pathway analysis results indicated the involvement of the proteins in cell growth, metabolism, immunomodulation, and extracellular matrix components. A functional analysis revealed the possible relations of the proteins to the local immune system, gametes maturation, fertilization, and early embryo development [44]. Several studies unveiled the molecular contents of oviductal EVs and were reviewed in Almiñana and Bauersachs [45]. In felines, EVs contain three-fold more proteins than in bovines and are enriched in proteins related to energy metabolism, membrane modification, and reproductive function. A total of 1511 protein groups were identified through ultraperformance liquid chromatography and tandem mass spectrometry (UPLC-MS/MS) [46]. Notably, a comprehensive analysis of bovine oviduct EVs revealed significant differences in hundreds of differentially expressed genes in frozen and fresh oviduct epitheliums [19].

**Figure 6:**
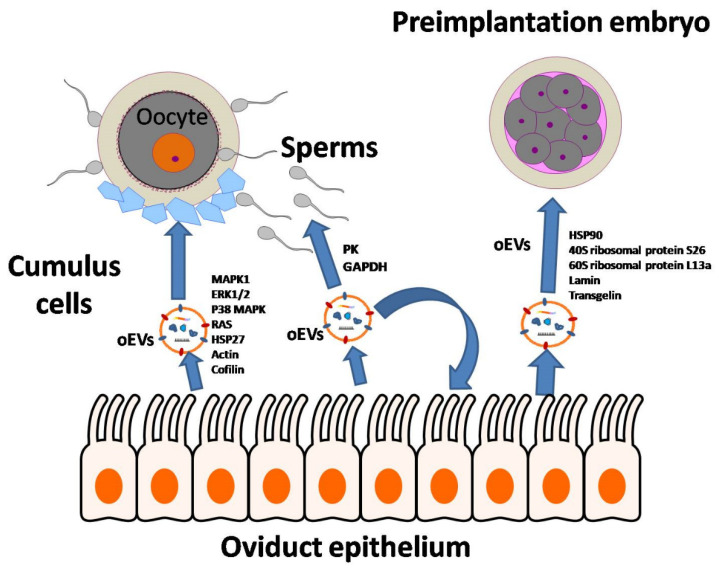
The proposed paracrine and juxtracrine actions of the oviductal cells-derived extracellular vesicles (OC-EVs) on the oocyte, sperms, embryos, and the oviduct cells. OC-EVs can mediate cargo transfer to the cells, such as the MAPK/ERK pathway, actin, and cofilin, for oocyte maturation and meiosis. Metabolic enzymes such as protein kinase (PK) and GAPDH can affect sperm metabolism as well. Moreover, some other proteins such as HSP90 and endoplasmic reticulum-related proteins can affect early preimplantation embryo development.

Oviduct EVs exert physiological actions on different spatial levels (Figure 6). Lee et al. [16] indicated that oviduct EVs upregulated the EGFR/MAPK signaling pathway in the canine cumulus cells on the level of oocyte maturation,. Moreover, oviduct EVs enhanced oocyte maturation and cumulus cell viability and proliferation, as well as reduced the production of reactive oxygen species and apoptotic rates. Additionally, according to our previous studies [16,17,28], we found that oviduct cells exposed to progesterone significantly improved the oocyte and cumulus cell development via the EGFR and MAPK(ERK)1/3 signaling pathways. Therefore, we assumed that progesterone would partially modify the protein content of EVs in this study. In the current study, the OC-EVs analysis identified several proteins that belong to ERK/MAPK signaling (Figure 5), such as MAPK1, ERK1/2, P38 MAPK, RAS, and HSP27. These pathways are involved in oocyte and cumulus proliferation and expansion [17,35,36,37,38]. Paradoxically, canine oviduct EVs at high concentrations might perturb oocyte maturation through targeting the TGFβ pathway via mir-375 [47]. On the embryonic level, oviduct EVs transferred mRNA and microRNA (miRNA) and altered the bovine embryo transcriptome [19]. In a murine model, supplementing an embryo transfer medium with oviduct EVs improved birth rates by preventing apoptosis and promoting differentiation [48]. On the oviduct level, a juxtracrine effect of oviduct EVs on the surrounding oviduct cells is also possible. An in vitro model showed that a culture with EVs derived from the oviductal mesenchymal cell line increased the number of ciliated cells in the Mullerian epithelial cell line, suggesting a juxtracrine/paracrine effect of oviduct cells in modulating their cell functions [49]. On the sperm level, as previously mentioned, EVs regulate sperm functions and capacitation [13,15,46,50], while there is scant information about the effects of oviduct EVs on canine sperm functions. A recent report showed that using dog oviduct EVs improved their post-thaw motility and prevented a premature acrosome reaction of red wolf spermatozoa [51].

Notably, several proteins were detected as associated with the actin cytoskeleton (Table S3), such as actin, cofilin, transgelin, and lamin. The cofilin-actin pathway is essential for meiotic development and cytokinesis during oocyte maturation [52]. A previous study suggested that actomyosin-cofilin pathways regulate meiotic spindle migration and cytokinesis during bovine oocyte maturation [53]. During cytokinesis, the intermediate filament vimentin (Table S3) contributes to the cleavage furrow, crucial for normal cell division. A slight distortion in the normal regulation of vimentin and other intermediate filament assembly/disassembly is associated with cytokinetic failure, aneuploidy, and binucleation, resulting in cell cycle distortion and cellular senescence [54,55].

Additionally, some metabolic enzymes were also detected, such as pyruvate kinase and glyceraldehyde-3-phosphate dehydrogenase (Table S3), which are key players in glucose metabolism in the cell [56]. A Gene Ontology analysis (Table S4) and IPA (Table S5) showed that OC-EVs contain proteins associated with different biological processes and canonical pathways involved in carbohydrate, lipid, and protein metabolism. Similarly, recent findings suggest that EVs regulate metabolism in COCs and/or embryos [11,23,39]. Besides, proteins associated with the pathways involved in embryonic development [57,58], such as actin, cyclin-dependent kinases, and several intermediate filaments, were also detected (Table S5).

The results also showed that OC-EVs contain numerous ribosome and RNA-binding proteins and other proteins involved in the process of protein synthesis, which may possess different RNAs to regulate gene expression and RNA degradation. They might transfer ribosomal constituents to the COCs and/or embryos (Figure 3) [59]. Moreover, protein processing in the rough endoplasmic reticulum is a fundamental process needed for cell survival in which the synthesis, folding, post-translational modification, transport, and sorting of proteins and some lipids occur [60,61]. Several proteins associated with rough endoplasmic reticulum functions were detected in the isolated EVs, such as endoplasmin (HSP90), 40S ribosomal protein S26, and 60S ribosomal protein L13a (Tables S1 and S2).

A functional analysis of the OC-EVs revealed processes related to cell death and survival (Table 1), indicating the possible role of EVs in regenerative effects on damaged cells of the oviductal canal, including the oocytes and/or the embryos [62]. Notably, studying the proteomics of oviductal EVs highlights the possible effects on embryonic development. Several studies reported the positive effects of oviduct-EVs on embryonic development in different species. In bovines, oviduct epithelial cell-derived EVs increased the embryo cell number (trophectoderm and inner cell mass) and the post-vitrification survival, in addition to the alteration of essential transcripts expression [14,63], rendering them superior quality. Moreover, in vitro-produced embryos were able to uptake in vivo oviduct EVs during the culture and increased the blastocyst rate, prolonged the embryo survival, and improved the embryo quality, and this was confirmed through the functional proteomics analysis [10].

Collectively, the extensive characterization of the protein cargo of OC-EVs revealed proteins that are associated with oocyte maturation and embryo development competence. Additionally, they may be associated with a variety of signaling processes that occur between the oocyte and cumulus cells, as well as cell death and survival. Our findings provide a strong basis for highlighting the potential function of OC-EVs as a paradigm for establishing a reliable system for in vitro oocyte maturation, in vitro fertilization, and the in vitro culture of preimplantation embryos in canine species.

## Figures and Tables

**Table 1:** The top ten biofunctions identified using the list of 398 common proteins from three biological samples of canine in vitro oviductal cell-derived extracellular vesicles.

Diseases or Functions Annotation Defined by Ingenuity Knowledge Base	Categories	*p*-Value	# Molecules
Initiation of translation of protein	Protein synthesis	2.81 × 10^−52^	49
Decay of mRNA	RNA damage and repair	8.95 × 10^−50^	44
Nonsense-mediated mRNA decay	RNA damage and repair	7.67 × 10^−48^	42
Translation	Protein synthesis	1.39 × 10^−42^	66
Metabolism of protein	Protein synthesis	7.94 × 10^−40^	112
Synthesis of protein	Protein synthesis	1.19 × 10^−39^	76
Translation of protein	Protein synthesis	4.66 × 10^−39^	62
Necrosis	Cell death and survival	5.92 × 10^−39^	171
Expression of protein	Protein synthesis	6.81 × 10^−37^	65
Cell death of osteosarcoma cells	Cell death and survival, and organismal injury	5.45 × 10^−32^	34

# Molecules: represent those identified in the list of 398 common proteins from three biological samples of canine in vitro oviductal cell-derived extracellular vesicles.

**Table 2:** The top ten canonical pathways identified using the list of 398 common proteins from three biological samples of canine in vitro oviductal cell-derived extracellular vesicles.

Canonical Pathway Defined by Ingenuity Knowledge Base	*p*-Value	# Ratio
EIF2 Signaling	5.01 × 10^−51^	0.25
Regulation of eIF4 and p70S6K Signaling	3.98 × 10^−27^	0.20
mTOR Signaling	1.26 × 10^−18^	0.13
Coronavirus Pathogenesis Pathway	7.94 × 10^−16^	0.15
Remodeling of Epithelial Adherens Junctions	3.16 × 10^−15^	0.24
Actin Cytoskeleton Signaling	3.16 × 10^−14^	0.11
Epithelial Adherens Junction Signaling	1.58 × 10^−13^	0.13
Integrin Signaling	1.00 × 10^−11^	0.10
Protein Ubiquitination Pathway	2.51 × 10^−11^	0.08
Glycolysis I	7.94 × 10^−11^	0.35

Ratio: # of common proteins from three biological samples of canine in vitro oviductal cell-derived extracellular vesicles/# of molecules that define the canonical pathway based on the Ingenuity Knowledge Base.

## Data Availability

The data presented in this study are available on request from the corresponding author. The data are not publicly available due to privacy/ethical restrictions.

## Supplementary Materials

The following are available online at https://www.mdpi.com/2076-2615/11/2/573/s1, Figure S1: SDS-PAGE protein separation. Figure S2: Common proteins identified in the three replicates of canine in vitro oviductal cell-derived extracellular vesicles that participated in the canonical pathway EIF2 signaling. Table S1: List of proteins identified in the canine in vitro oviductal cell-derived extracellular vesicles from three different individuals. Table S2: List of common proteins identified in the three replicates of the canine in vitro oviductal cell-derived extracellular vesicles. Table S3: Top 20 of the identified 398 shared proteins from three biological samples of the canine in vitro oviductal cell-derived extracellular vesicles. Table S4: Gene Ontology (GO) and PANTHER analysis of the protein clusters of the in vitro oviductal cell-derived extracellular vesicles. Table S5: Core analysis generated by an Ingenuity Canonical Pathways analysis using the list of common proteins of the in vitro oviductal cell-derived extracellular vesicles.

## Abbreviations

EVs	Extracellular vesicles
OC-EVs	In vitro oviductal cell-derived extracellular vesicles
COCs	Cumulus–oocyte complexes
EGFR	Epidermal growth factor receptor
MAPK	Mitogen-activated protein kinase
LC-MS/MS	Liquid chromatography-tandem mass spectrometry
NTA	Nanoparticle tracking analysis
HSP	Heat shock protein
GO	Gene Ontology
BPs	Biological processes
MFs	Molecular functions
CCs	Cellular components
IPA	Ingenuity Pathway Analysis
FBS	Fetal bovine serum
SDS-PAGE	Sodium dodecyl sulfate-polyacrylamide gel electrophoresis
TFA	Trifluoroacetic acid
FDR	False Discovery Rate
