# Supplementary material for: Comprehensive Proteomics Analysis of In Vitro Canine Oviductal Cell-Derived Extracellular Vesicles
Source: Animals (Basel). 2021 Feb 23;11(2):573. doi: 10.3390/ani11020573 (PMC7926305; doi:10.3390/ani11020573)
Supplement: Supplementary file 1 [file animals-11-00573-s001.zip › Figure S2.docx]

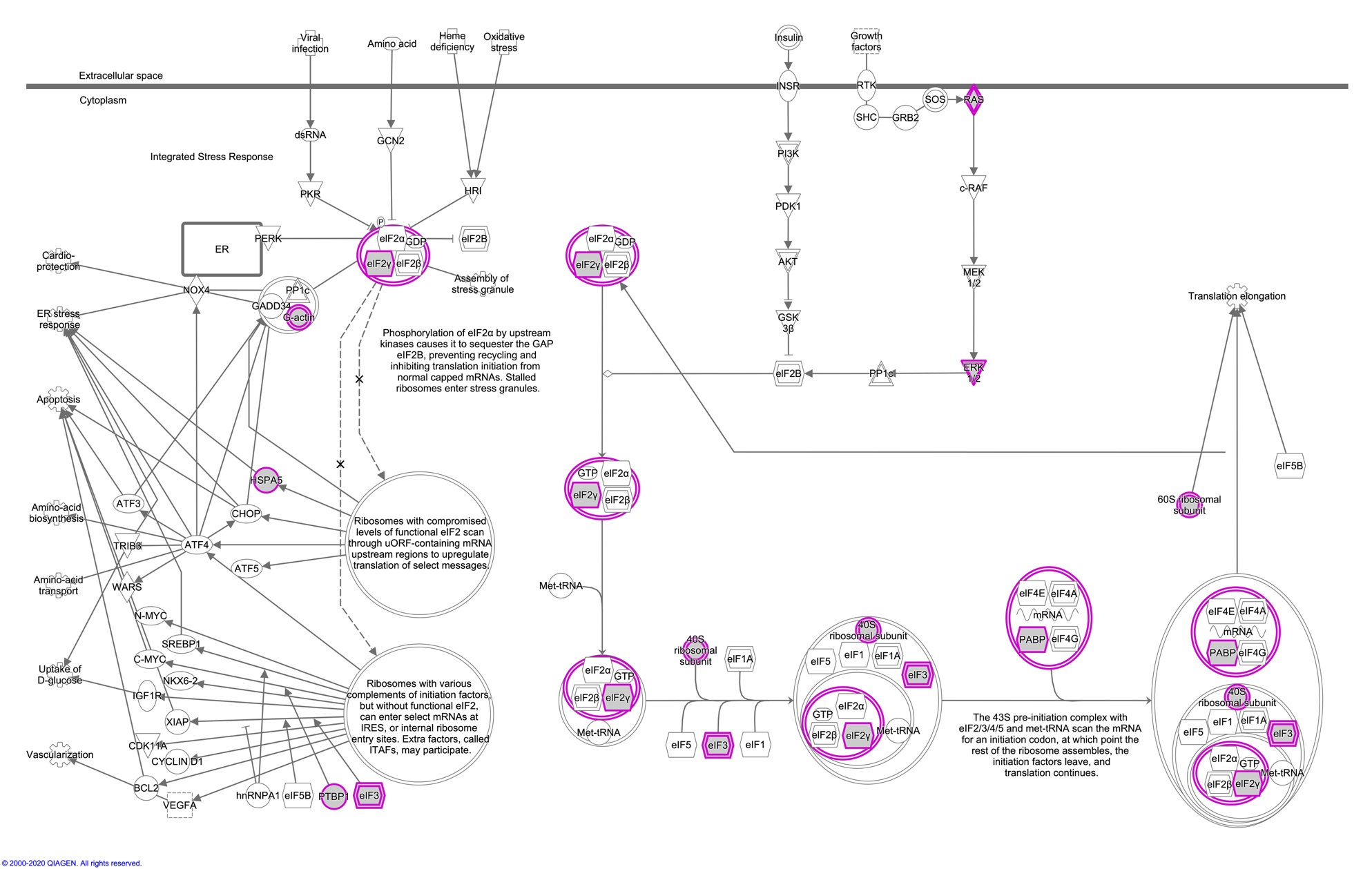


**Figure S2.** Common proteins identified in the three replicates of canine oviductal EVs that participate in the canonical pathway (CP) EIF2 signaling. This CP is defined by the Ingenuity Knowledge Base. The grey molecules highlighted with pink were identified in three biological samples of canine oviduct-derived exosomes.
