# Supplementary material for: Comprehensive Proteomics Analysis of In Vitro Canine Oviductal Cell-Derived Extracellular Vesicles
Source: Animals (Basel). 2021 Feb 23;11(2):573. doi: 10.3390/ani11020573 (PMC7926305; doi:10.3390/ani11020573)
Supplement: Supplementary file 1 [file animals-11-00573-s001.zip › Table S3 (List of top 20 common proteins).docx]

**Table S2.** Top 20 of identified 398 shared proteins from three biological samples of canine oviduct-derived exosomes.

| **Description of common proteins** | **Mol %** | **Accessions** |
| --- | --- | --- |
| Vimentin | 13.93 | F1PLS4 |
| Annexin A2 | 3.58 | Q6TEQ7 |
| Transgelin | 3.11 | E2RIF3 |
| Histone H4 | 2.61 | F2Z4N2 |
| Transgelin | 1.63 | F1P6P2 |
| Peptidyl-prolyl cis-trans isomerase | 1.36 | F1PK62 |
| Actin, Alpha | 1.28 | F2Z4N7 |
| Tubulin beta chain | 0.88 | L7N0I7 |
| Tubulin beta chain | 0.87 | E2QSF4 |
| Cofilin 1 | 0.76 | F1PQN5 |
| Serpin family H member 1 | 0.74 | E2RHY7 |
| Ribosomal protein S19 | 0.72 | J9P425 |
| Lamin A/C | 0.69 | J9NSW5 |
| Fatty acid binding protein 3 | 0.69 | E2R507 |
| Tubulin beta chain | 0.65 | E2RFJ7 |
| Annexin | 0.60 | F1P6B7 |
| Pyruvate kinase | 0.58 | F1PHR2 |
| Glyceraldehyde-3-phosphate dehydrogenase | 0.57 | F1PTZ9 |
| 40S ribosomal protein S18 | 0.38 | Q5TJE9 |
| Ras-related protein Rab-1A | 0.34 | P62822 |
