# Supplementary figures and images for: Comprehensive Proteomics Analysis of In Vitro Canine Oviductal Cell-Derived Extracellular Vesicles
Source: Animals (Basel). 2021 Feb 23;11(2):573. doi: 10.3390/ani11020573 (PMC7926305; doi:10.3390/ani11020573)

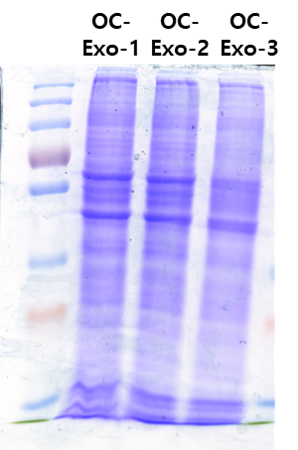

Supplement: Supplementary file 1 [file animals-11-00573-s001.zip › Figure S1.tif]
